# Pseudo-Orthostatic Tremor in Graves’ Disease: A Possible Early Sign of Parkinsonism?

**DOI:** 10.5334/tohm.924

**Published:** 2024-07-24

**Authors:** Davide Comolli, Simone Regalbuto, Sebastiano Arceri, Giuseppe Trifirò, Alessandra Calculli, Carlo Fazio, Piergiorgio Grillo, Massimiliano Todisco, Antonio Pisani

**Affiliations:** 1 Department of Brain and Behavioral Sciences, University of Pavia, Pavia, Italy; 2 IRCCS Mondino Foundation, Pavia, Italy; 3 Nuclear Medicine Unit, Istituti Clinici Scientifici Maugeri, IRCCS, Pavia, Italy; 4 Marlene and Paolo Fresco Institute for Parkinson’s and Movement Disorders, NYU Langone Health, New York, New York, USA

**Keywords:** Pseudo-orthostatic, Tremor, Autoimmune thyroiditis, Parkinsonism, Abnormal DaT-SPECT

## Abstract

**Background:**

Pseudo-orthostatic tremor is a hyperkinetic movement disorder usually associated with other neurological comorbidities, mainly Parkinson’s disease.

**Case report:**

A 65-year-old male presented with unsteadiness and leg tremor while standing. Electrophysiological evaluation confirmed the presence of pseudo-orthostatic tremor. Blood test showed an undiagnosed Graves’ disease. A complete remission of tremor was achieved with methimazole. Dopamine transporter scintigraphy showed a mild reduction of the striatal binding, bilaterally.

**Discussion:**

Graves’ disease can be associated with pseudo-orthostatic tremor. Thyroid function should be assessed in patients complaining of unsteadiness. The causative role of hyperthyroidism in determining dopaminergic degeneration and uncovering subclinical parkinsonism warrants further investigations.

## Introduction

Orthostatic tremor (OT) is a rare neurological disorder presenting with an unsteadiness while standing that disappears when the patient walks, sits or lies down. The diagnosis is confirmed by an EMG assessment which shows a high-frequency tremor of between 13 and 18 Hz in the lower limbs whilst in a standing position [1]. Although this condition occurs in isolation in approximately two-thirds of cases (primary OT) associations with other neurological disorders are observed in one third of patients (primary OT plus) [2]. In cases when the tremor frequency is lower, the disease is identified as a pseudo-orthostatic tremor (PsOT), previously known as slow OT. PsOT is more frequently, in approximately two-thirds of cases, associated with other neurological disorders: parkinsonism alone accounts for one-third of cases and other frequent disorders include cerebellar ataxia and dystonia [3]. Graves’ disease (GD) is an autoimmune disorder where the production of anti-TSH receptor antibodies leads to overstimulation of the thyroid and clinical hyperthyroidism. Although the literature on thyroid dysfunction and movement disorders includes cases of OT/PsOT, diagnosed in concomitance with GD, it is not yet clear if and how these two entities are linked to each other. Here, we present a new case of PsOT in GD, with suggestive neuroimaging, which provides useful insights.

## Case description

A 65-year-old male was admitted to our clinic for lumbar pain associated with unsteadiness and leg tremor while standing. The latter symptom started three months prior and had progressively worsened. He also reported paraesthesia in both hands. Previous spine MRI scans showed only minimal cervical and lumbar discal protrusions. The patient had an unremarkable past medical history and was not prescribed any medication. The neurological examination showed a fine postural tremor of both hands and a visible slow-frequency tremor of the legs, only presenting during maintenance of an upright posture, with a lack of balance in the Romberg position. The patient also reported a significant weight loss (of 5–6 Kg) in the past four months.

**Figure 1:**
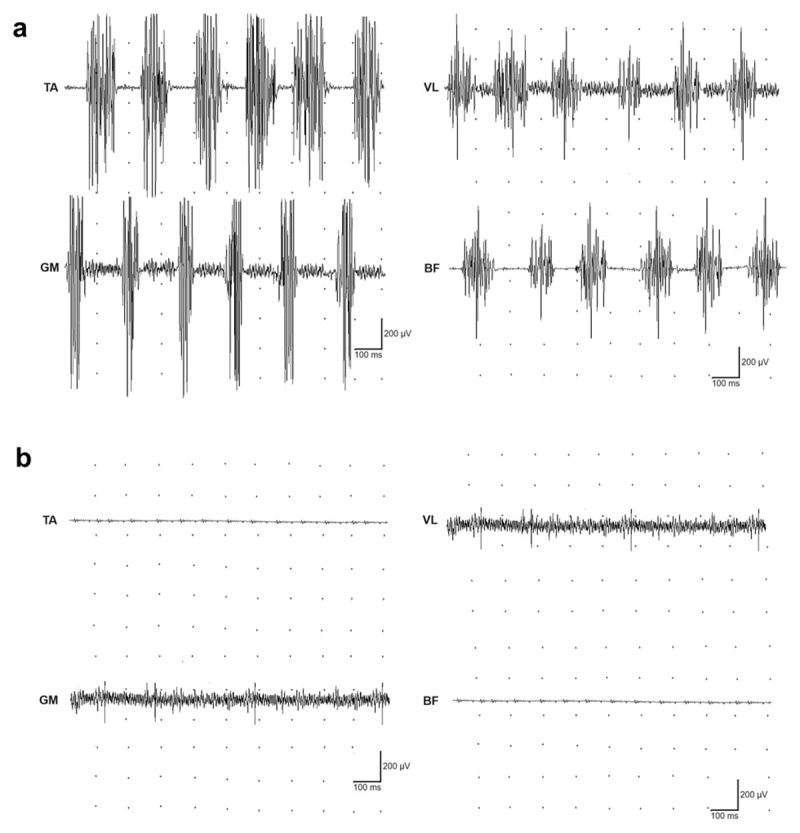
**EMG of the lower limb muscles before (a), and nine months after treatment introduction with methimazole (b)**. The traces show PsOT at 6 Hz, exclusively when the patient was asked to keep the standing position (tremor was absent at rest or at Mingazzini II position). Tremor was characterized by alternated activation among the examined pairs of antagonist muscles (TA, tibialis anterior/GM, gastrocnemius medialis; VL, vastus lateralis/BF, biceps femoris). Only recordings from the right leg are shown, since findings from the left leg were similar.

After excluding a polyneuropathy or other peripheral nervous system disorder, an EMG recording with concentric needle electrodes found a 6-Hz tremor at the level of both the distal and proximal muscles of the lower limbs, only presenting when the patient was asked to stand up (Figure 1a). Such elements were compatible with a diagnosis of PsOT.

Blood tests showed an unknown hyperthyroidism (TSH: 0.005 mU/L; T3: 16.4 pmol/L; T4: 47 pmol/L). No other symptoms of hyperthyroidism were reported, except the aforementioned weight loss. Detection of the anti-TSH receptor antibody (5.86 U/L) confirmed an undiagnosed case of GD as the cause of the hyperthyroidism. As a result, treatment with methimazole was initiated. At a two-month follow-up, blood tests indicated normal thyroid function, along with the complete resolution of both postural instability and tremor. Nine months after diagnosis, symptoms were still completely controlled with methimazole and a second EMG assessment showed no tremor in the lower limbs while standing (Figure 1b).

**Figure 2:**
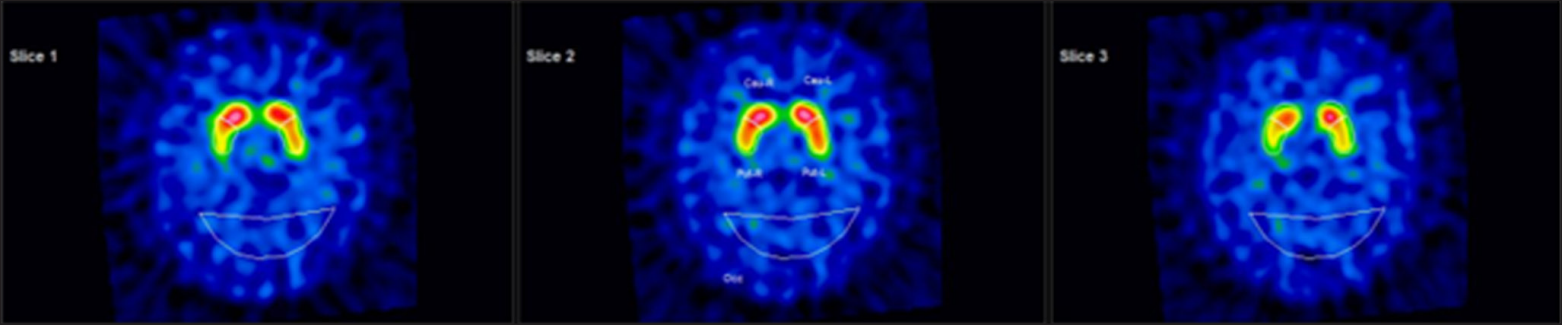
**Qualitative and semiquantitative analysis of [**^123^**I]-ioflupane SPECT executed nine months after treatment introduction with methimazole**. Values show a mild to moderate hypocaptation in the caudate nucleus, more prominent on the left side, and in the putamen, bilaterally. Captation ratios for left caudatum/occipit 2.70 [3.64+/–0.50] and right caudatum/occipit 2.81 [3.64+/–0.50]; Captation ratios for left putamen/occipit 2.42 [3.02+/–0.56] and right putamen/occipit 2.40 [3.02+/–0.56]. Total striatum/occipit ratio 2.58 [3.23+/–0.50]; putamen/caudatus ratio 0.87 [0.83+/–0.07].

In consideration of the well-documented association between PsOT and other neurological disorders, in particular degenerative parkinsonisms, we also performed both qualitative and semiquantitative analyses of dopamine transporter imaging by means of [^123^I]-ioflupane SPECT. This indicated a mild bilateral reduction of the striatal binding, with homogeneous involvement of putamen and caudate nucleus (Figure 2). No signs of bradykinesia or rigidity were detected by neurological examination. It is of note that the patient referred to a history of hyposmia, but with no constipation or other symptoms compatible with Rapid Eye Movement Sleep Behavior Disorder (RBD).

## Discussion

The overlap between thyroid dysfunction and movement disorders is a matter of interest in daily clinical practice, being the first condition highly prevalent in the general population. Tremor, for example, is a classical presentation of hyperthyroidism, but few cases of chorea have also been reported. On the other hand, papillary thyroid cancer and Hashimoto disease, both resulting in hypothyroidism, occasionally include dystonia, chorea and myoclonus in their clinical spectrum. Although there is uncertainty about the biological mechanisms linking thyroid and movement disorders, the hormone changes and immune dysregulation which are typical of thyroiditis may play a crucial role. Genetic complications impacting on the thyroid-basal ganglia interaction may also play a role, as observed in the Brain-Lung-Thyroid Syndrome, linked to mutations in *NKX2-1* [4].

There are only one case of OT and two cases of PsOT reported in literature in association with GD (Table 1), other than a recent report of acute-onset unsteadiness following thyrotoxicosis due to a cerebral angiography with subsequent self-resolution. The clinical characteristics of this case were compatible with OT, but were not confirmed by electrophysiological examination [5]. In all of the known cases of OT or PsOT which were linked to GD, symptoms resolved with the normalization of thyroid function. In all of these cases, structural imaging investigations (brain and spine MRI) were unremarkable. This is a common occurrence in PsOT, where the focal lesions are present in only a small percentage of patients (about one-fifth of cases, mainly focal lesions of cerebellum, medulla, pons or upper spinal cord) [3]. An exception is observed with molecular brain imaging, since dopamine transporter scintigraphy frequently shows hypocaptation in the basal ganglia of patients with concomitant parkinsonism, in particular Parkinson’s disease (PD), which is commonly associated with PsOT. However, dopamine transporter scintigraphy was not performed in any of the cases reported in association with GD. In our patient, despite the complete absence of any motor symptom related to PD, the mild reduction of striatal binding suggested a concomitant subclinical degenerative parkinsonism.

**Table 1:** **Reported cases of OT or PsOT associated with GD**. All other instrumental examinations performed other than EMG and thyroid function tests were unremarkable.


AUTHORS	PATIENT AGE AND SEX	NOTABLE CLINICAL FEATURES	TREMOR FREQUENCY	OTHER INSTRUMENTAL EXAMINATION PERFORMED	TREATMENT

Lin et all. [12]	26 years old male	None	8–9 Hz PsOT	Brain, thoracic and lumbosacral spine MRI	Methimazole

Mazzucchi et all. [13]	70 years old female	Tetrahyperreflexia inability to perform tandem gait	8 Hz PsOT	Brain, cervical and dorsal spine MRI Motor-evoked potential	Methimazole

Tan et all. [14]	50 years old female	Tremor present only in the right leg	14–16 Hz OT	Brain and lumbosacral spine MRI	Carbimazole


A possible common denominator between thyroid dysfunction and striatal impairment may be found in the existing connection between hypothalamic–pituitary–thyroid axis and dopamine. Hypothalamic hormone TRH and thyroxine interact with the dopamine system, with opposite effects. The first activates pre-synaptic dopaminergic neurons and stimulates dopamine release, the latter, instead, increases the degradation of the neurotransmitter, alters the sensitivity of DA receptors [6], and regulates the activity of some striatal proteins, such as the small GTP-binding protein Rhes which is highly expressed in the dopaminoceptive striatal GABAergic projection neurons [7]. It is reported, indeed, that hyperthyroidism worsens fluctuations and motor symptoms (not only tremors but also akinesia and rigidity) in PD patients, who later improve when euthyroidism is restored [4]. In GD, where the TRH is typically reduced and thyroid overstimulation results in thyrotoxicosis, the dopamine system would be overall inhibited.

It is likely that a functional alteration of neurotransmission may not be the only link between GD and striatal impairment. A further mechanism might be related to the dysregulation of the immune system and neuroinflammation, conditions which play a crucial role in both the susceptibility to and the progression of PD [8]. In fact, it has been observed that GD patients have a higher incidence of PD than the general population [9], a point which suggests that subjects with autoimmune comorbidities may be prone to developing neurodegenerative diseases. Another possible mechanism is mediated by an oxidative stress increase in neurons, since hyperthyroidism is associated with an increase in cellular metabolism, especially in mitochondria, a process that is known to be involved in the pathogenesis of PD [10]. However, the possibility that directly or indirectly, thyroid dysfunction may be a contributory factor in the neurodegenerative processes is purely speculative, and will require further experimental and clinical demonstration.

It is known that the tremors associated with hyperthyroidism resemble enhanced physiological tremors, as both are caused by the activation of peripheral β2-adrenergic receptors. This activation is attributed to both the increased catecholamine content in anxiety and an increased number of β-adrenergic receptors mediated by thyroxine in thyrotoxicosis. This type of tremor is characterized by a high frequency and low amplitude postural tremor, predominantly affecting the hands. In our case, the tremor had a lower frequency, mainly involved the legs, and most importantly, was present only when the patient was standing still [11].

To confirm the hypothesis of a pathophysiological linkage between hyperthyroidism and degenerative parkinsonism, the patient will continue to undergo neurological follow-up examinations for a prompt detection of motor symptoms which may indicate the future development of clinical parkinsonism. It is interesting that, when asked, the patient also reported a history of hyposmia that was never investigated, but no other prodromal or non-motor sign of parkinsonism.

This report emphasizes the importance of thyroid function screening in patients complaining of unsteadiness compatible with OT or PsOT, a disease that is largely underdiagnosed. Since GD is an entirely treatable condition, early identification could guarantee the complete remission of PsOT. To conclude, our case suggests that this might be considered a pseudo-OT, caused by a possible pre-motor PD, and eventually unmasked by GD.
